# Brain mechanisms for processing caress-like touch in skin-picking disorder

**DOI:** 10.1007/s00406-023-01669-9

**Published:** 2023-08-23

**Authors:** Anne Schienle, Carina Schlintl, Albert Wabnegger

**Affiliations:** Clinical Psychology, University of Graz, BioTechMed, Universitätsplatz 2/III, 8010 Graz, Austria

**Keywords:** Affective touch, Skin-picking disorder, Somatosensory integration, Attention

## Abstract

Skin-picking disorder (SPD) is characterized by repetitive touching and picking of one’s skin. The picking is typically experienced as pleasant although this behavior leads to tissue damage. The present functional magnetic resonance imaging (fMRI) study investigated how individuals with SPD react to caress-like touch, which stimulates C-tactile afferents. A standardized touch procedure was used. Seventy females with a primary diagnosis of SPD and 62 healthy females received CT-optimal brushing of their forearms (3 cm/s) and non-optimal brushing (30 cm/s) during an fMRI session. The two types of tactile stimulation were rated according to pleasure, arousal, and urge to pick one’s skin. Relative to healthy controls, patients with SPD showed greater activation in parietal regions (supramarginal/angular gyrus) during CT-optimal touch. Moreover, the deactivation of the middle/ inferior frontal cortex displayed by control participants was absent in the SPD group. Being touched was rated as less pleasant, more arousing, and elicited a greater urge to perform skin-picking in participants with SPD. The mentioned frontal and parietal brain regions are sources of attentional control. They are involved in integrating somatosensory information and switching attention between external/internal stimuli. The present study adds to the limited database on the dysfunctional processing of touch in SPD. This study was preregistered on the German Clinical Trials Register (DRKS00022123, June 8th, 2020).

**Supplementary Information:**

The online version contains supplementary material available at 10.1007/s00406-023-01669-9.

## Introduction

Repetitive touching, rubbing, scratching, picking at, and digging into one’s skin are symptoms of skin-picking disorder (SPD), also referred to as excoriation disorder or dermatillomania [1]. This behavior can lead to tissue damage, distress, and functional impairment. SPD is a common disorder (estimates of prevalence range between 2–3% in the general population) that affects more females than males [2].

Individuals with SPD typically pick at minor skin irregularities (e.g., pimples, calluses, scabs), which are identified by visual inspection and tactile checking (repetitive touching). Picking episodes are often preceded by negative affective or somatic states (e.g., feelings of anger, and bodily tension) [3]. During these episodes, most patients experience the picking as pleasant since it leads to a reduction of tension, and is perceived as soothing or calming, or even meditative/trance-like [3, 4]. The positive valence of skin-picking episodes is surprising since the excessive manipulation of the skin causes tissue damage which would generally be experienced as unpleasant. The phenomenology of SPD described here points to possible dysfunctions in the processing of tactile information.

The human touch system can be divided into a ‘fast’ system with discriminative functions (e.g., touch localization) and a ‘slow’ system with social-affective functions [5]. The slow system is responsive to caress-like touching of the skin, which activates specific nonmyelinated nerve fibers, so-called C-tactile (CT) afferents. Discriminative touch is mediated via myelinated peripheral A-beta fibers [5].

The most widely used method for studying responses to CT-optimal touch consists of administering soft brush strokes to the forearms of the participants with velocities between 1 and 10 cm/s [6]. This type of tactile stimulation is typically perceived as pleasant and elicits activation in brain regions such as the primary and secondary somatosensory cortex, prefrontal cortex regions, and the insula [7–9].

Deficits in caress-like touch processing have been documented for several mental disorders that are accompanied by difficulties in social interactions, such as autism, autism spectrum disorders, and borderline personality disorder [10–12]. Moreover, childhood neglect/abuse is associated with the reduced experience of the pleasantness of affective touch in adulthood [13]. Studies on touch processing in SPD are still lacking, with one exception. A functional magnetic resonance imaging (fMRI) study [14] provided the first evidence for the atypical processing of self-touch in SPD. The participants were instructed to either caress or scratch a small skin area on their forearms. Compared to healthy controls, participants with SPD showed less activation in frontal and primary/secondary somatosensory cortex regions during caressing relative to scratching. This finding hinted at reduced sensitivity of soft touch in patients with SPD.

Other studies have identified tactile over-responsivity in individuals who display body-focused repetitive behaviors (BFRBs, such as skin-picking and hair-pulling [15–17]. These studies showed that people with pathological skin-picking overreact to external (environmental) stimuli, including soft-touch (e.g., touching ordinary textures of cloth or food). In line with the concept of tactile over-responsivity, Schienle et al. [18] demonstrated that four weeks of daily soft-touch training for patients with SPD (guided soft brushing of selected skin regions) decreased brain activity in the parietal operculum (PO) and supramarginal gyrus (SMG) in response to soft brushing of the skin. Both regions are involved in the processing of different touch characteristics (sensory, affective, and action-dependent; e.g., [19–21]). The reduction in brain activation within the PO and SMG due to the training was associated with increased ratings for the pleasantness of soft touch.

In the present fMRI study, participants with SPD and healthy females received slow/soft brushing (CT-optimal) and fast/soft brushing (control condition) to their forearms. The participants rated the two conditions according to pleasure, arousal, and urge to pick their skin. To the best of our knowledge, responses to touch by others have not been compared between people with and without SPD. Previous findings on tactile processing in SPD have pointed to both hyposensitivity to soft touch [14].; concerning self-touch) as well as hypersensitivity (e.g., [17]; concerning touching soft objects). Therefore, the analysis approach for the present study was exploratory. It was investigated whether participants with/without SPD would differ in their affective ratings for slow/soft touch and associated activity in functionally specialized brain areas: somatosensory regions, the insula, and prefrontal cortex regions. Additionally, functional connectivity between the selected brain regions was analyzed and compared between the two groups (via the generalized psychophysiological interaction approach). The two analysis methods can help to understand how localized hypo/hyper-activity, as well as hypo/hyper-connectivity, may contribute to altered touch processing in SPD.

## Methods and materials

### Participants

Seventy female patients with a primary DSM-5 diagnosis [1] of skin-picking disorder (SPD group) and 62 healthy females (Control group; CG) participated in this study. The two groups did not differ in mean age (M_SPD_ = 25.57 years, SD = 6.82; M_CG_ = 23.87 years (5.59); t(130) = − 1.55 (p = 0.123), years of education (≥ 12 years): SPD = 97%; CG = 97%, and handedness (> 80% right-handedness in both groups; χ^2^ = 2.77, p = 0.250).

Exclusion criteria for the SPD group were diagnoses of major depression with severe symptoms, substance abuse/ dependence, borderline personality disorder, psychosis, and dermatological conditions (e.g., scabies, psoriasis, atopic dermatitis). Exclusion criteria for the control group were reported diagnoses of mental disorders, dermatological conditions, and psychotropic medication. The sample was restricted to females because of gender differences concerning the prevalence of SPD and affective touch processing.

A statistical power analysis indicated that for an effect size of f = 0.16, with a power of 0.95 and an alpha level of 0.05 for a mixed-model analysis of variance (two between-subjects factors, two within-subjects factors; correlation between repeated measures: 0.5) 130 participants would be needed.

### Procedure

The study complied with all relevant ethical guidelines and regulations involving human participants and was approved by the ethics committee of the University of Graz (Austria; GZ 39/29/26 ex 2018/19). All participants provided informed consent before participating. This study was preregistered on the German Clinical Trials Register (DRKS00022123, June 8th, 2020). Individuals were recruited via the outpatient clinic of the university and social media. The participants completed the following questionnaires and tests:

- The Skin-Picking Scale revised ([22] SPS-R; Cronbach’s alpha in the present sample α = 0.96) is a self-report questionnaire to assess the severity of skin-picking symptoms. The scale contains eight items covering the following domains: (a) frequency of the urge to pick, (b) intensity of the urge to pick, (c) time spent picking, (d) control over picking, (e) functional impairment, (f) emotional distress, (g) avoidance behavior, (h) skin damage due to picking. Each item is rated on a 5-point scale from 0 (none) to 4 (extreme). We computed a sum score.
- The Milwaukee Inventory for the Dimensions of Adult Skin Picking ([23]; MIDAS; α = 0.84) is a self-report measure to assess focused and automatic skin picking. The two subscales comprise six items each (focused: e.g., “I pick my skin when I am experiencing a negative emotion such as stress, anger, frustration, or sadness”; automatic: e.g., “I am usually not aware of picking my skin during the picking episode”). Each item of the MIDAS is rated on a five-point scale ranging from 1 (= “not true for any of my skin picking”) to 5 (= “true for all of my skin picking”).
- The Skin Picking Impact Scale ([24] SPIS; Cronbach’s α = 0.94) is a 10-item self-report scale designed to assess the psychosocial impact of SPD symptoms (e.g., “I feel unattractive because of my skin picking”) as well as social interference due to the disorder (e.g., “My relationships have suffered because of my skin picking”). Items are rated on a 6-point scale from 0 (not) to 5 (extremely) to compute a total mean score.
- As a measure of tactile acuity, the two-point discrimination test was conducted. The W54670 (Baseline) sensitivity tester (Fabrication Enterprises Inc.; model number: 12–1492) was used to assess the ability of the participants to discern two nearby points (distances ranging between 2–5 mm) at the tip of their dominant index finger. The test used a forced-choice technique; the two-point threshold was defined as the smallest distance at which 7 out of 10 tactile stimulations were correctly identified. This test was chosen as an index of discriminative touch processing.

All patients with SPD were interviewed by a board-certified clinical psychologist with a standardized diagnostic interview for mental disorders [25] with additional questions concerning skin-picking symptoms according to DSM-5 (based on the Yale Brown Obsessive Compulsive Scale Modified for Neurotic Excoriation; [26]).

### Experimental design

During the fMRI recording, tactile stimulation was administered by a female research assistant, who used a hand-held soft boar bristle brush (Bipa essentials). The experimenter had been trained to deliver strokes at a constant pressure (220 mN) with a specific velocity. The velocity of touch was guided by a metronome (via headphones). CT-optimal touch had a velocity of 3 cm/s (stroking in proximal to distal direction, 8 cm region), whereas CT-nonoptimal touch had a velocity of 30 cm/s. Each brushing condition lasted for six seconds and was repeated 12 times during the experiment. The conditions were interspersed by rest periods (no brushing) lasting for 12 s. The sequence of the brushing conditions was randomized.

**Fig. 1:**
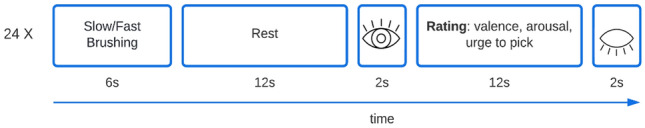
Experimental procedure

After each condition, the participants rated their emotional state (pleasantness, arousal) on a 9-point scale (9 = very pleasant, very aroused) and the urge to pick their skin (9 = maximal urge). A first signal tone (presented for 2 s) indicated opening the eyes and responding to the visually presented rating scales (12 s). Participants gave their ratings verbally via a scanner-suitable microphone. A second signal tone (2 s) indicated closing the eyes for the subsequent brushing condition (Fig. 1).

### fMRI recording

The MRI session was conducted with a 3 T scanner (Vida, Siemens, Erlangen, Germany) with a 64-channel head coil. Functional runs were acquired using a T2*-weighted multiband EPI protocol (number of slices: 58, interleaved, flip angle = 82°, slice thickness: 2.5 mm; slice spacing: 3 mm; TE = 0.03; TR = 1800 ms; multi-band accel. factor = 2; acquisition matrix: 88; in-plane resolution = 2.5 × 2.5 × 2.5 mm). Structural images were obtained using a T1-weighted MPRAGE sequence (voxel size: 1 × 1 × 1 mm; acquisition matrix: 224, slice thickness: 1 mm, TE = 0.00236, TR = 1600 ms; flip angle = 9°). All analyses were conducted with SPM12 (version: 7487; Wellcome Department of Cognitive Neurology, London). Functional images were first realigned and unwarped by registering images to the first image with a 2nd Degree B-Spline interpolation. Afterward, images were slice-time corrected (middle reference slice). Subsequently, individual anatomical images were segmented into grey matter, white matter, and cerebrospinal fluid, which were further used to create a skull-stripped image. Realigned/unwarped and slice-time corrected images were then co-registered to the skull-stripped image using the normalized mutual function. Forward deformations were used to normalize functional images (voxel size 2 × 2 × 2 mm), which were finally smoothed with a Gaussian full-width at half maximum (FWHM) of 8 mm.

For the first-level analyses, the following parameters were included in the design matrix (CT-optimal touch, CT-nonoptimal touch, rating scale). Further, we extracted six components of WM and CSF and calculated the framewise displacement for individual time series with the physio toolbox. Together with the six motion parameters these parameters were introduced as regressors of no interest into the design matrix. Based on the framewise displacement analysis, five participants had to be excluded from further fMRI analysis because more than 25% of the individual volumes exceeded the predefined threshold (0.5 mm). We compiled ‘CT-optimal–nonoptimal’ as the contrast of interest to model event-related responses by the canonical hemodynamic response function. Data were high-pass filtered (175 s) and serial correlations were accounted for by using an autoregressive AR(1) model.

### Statistical analysis

#### Self-report data

Questionnaire/ test scores were compared between the two groups via t-tests. A mixed-model analysis of variance (ANOVA) tested the effects of Group (SPD, Control) and Type of Touch (CT-optimal/nonoptimal) on self-reported arousal, valence, and urge to pick one’s skin during tactile stimulation. Effect sizes are expressed by generalized eta squared (η^2^G). All statistical analyses were performed with Jamovi (version 2.2.2.0).

#### Brain imaging data

The contrast of interest was compared between groups (SPD vs. Control) using a two-sample t-test. Results were assessed by using a cluster-building threshold of 0.005 (uncorrected) with at least three contiguous voxels. For inferences, we considered whole-brain voxel peaks as statistically significant when p corrected for family-wise error (FWE) was below 0.05. Based on previous findings [14, 18], region of interest (ROI) analyses were carried out for the insula, somatosensory cortex regions (e.g. supramarginal gyrus, parietal operculum), and prefrontal cortex regions (e.g., inferior frontal gyrus). Masks for the ROI analyses were taken from the Harvard–Oxford probability atlas (threshold: 25%). All fMRI analyses were performed with SPM (v7484) implemented in Matlab R2019b.

#### Exploratory connectivity analyses

The brain regions that differed in activation between the SPD group and the Control group (contrast: CT-optimal–nonoptimal touch) were selected as seeds/regions of interest (IFG, MFG, SMG, ANG) for generalized PsychoPhysiological Interactions analyses [27]. For the seed regions, a 6-mm sphere was built around the activation peak. The extracted time course for the specific seed region was then used as an additional regressor in the general linear model analysis. Regions of interest were the same as in the activity analysis.

#### Exploratory correlation analyses

Correlations were computed separately for the SPD group and the Control group to identify possible associations between ROI activity and averaged ratings for valence, arousal, and urge to pick one’s skin.

## Results

### Clinical interview

Reported symptom duration was on average M = 13.8 years (SD = 7.07; symptoms since childhood: 32%; puberty: 57%, adulthood: 11%). Duration of picking per day was M = 2.5 h (SD = 2.1; range: 1–12). All participants with SPD reported both focused as well as automatic skin-picking at several sites of the body (predominantly hands, arms, and face).

Comorbid mental disorders were diagnosed in 47% of the patients. The diagnoses included anxiety disorders (generalized anxiety disorder, panic disorder, specific phobia; 36%); depression (mild to moderate symptoms; 4%); obsessive–compulsive disorder (3%), and eating disorders (6%). None of the patients had a diagnosis of trichotillomania. Three patients with SPD (4%) were taking psychotropic medication (selective serotonin reuptake inhibitors; n = 2; serotonin-norepinephrine reuptake inhibitors; n = 1).

### Questionnaires

Questionnaire scores (means, standard deviations) are depicted in Table 1. The SPD group obtained higher scores on the disorder-specific scales than the Control group.

**Table 1:** Comparison of questionnaire scores between the SPD group and the Control group: means, standard deviations (SD), and confidence intervals (CI)

	SPD group (n = 70)	Control group (n = 62)	t_82.56–129.16_
	M (SD) [CI]	M (SD) [CI]	
SPS_R	16.36 (2.88) [15.69–17.00]	1.42 (1.95) [0.98–1.90]	− 35.16***
SPIS	3.64 (0.80) [3.46–3.83]	1.08 (0.24) [1.02–1.15]	− 25.54***
MIDAS_focused	23.61 (3.93) [22.76–24.52]	8.68 (3.89) [2.68–4.90]	− 21.92***
MIDAS_automatic	18.80 (4.79) [17.52–20.08]	14.84 (3.91) [14.00–15.77]	− 5.23***

*SPS_R* Skin-picking scale revised, *SPIS* Skin-picking impact scale, *MIDAS* The Milwaukee Inventory for the Dimensions of Adult Skin Picking, *CI* confidence intervals are bias-corrected and accelerated (sample = 1000); ***p < 0.001.

### Touch ratings

**Fig. 2:**
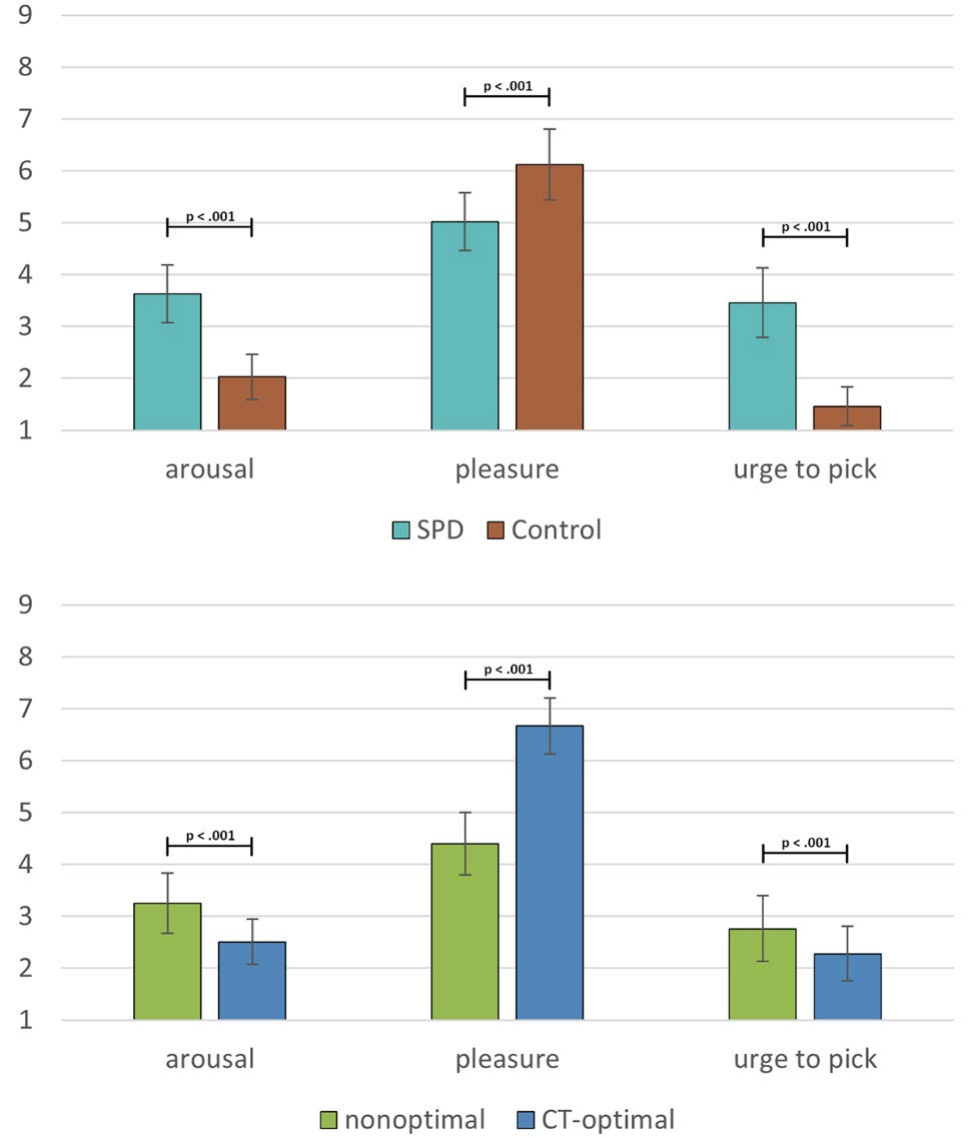
Affective ratings (means, 95% CIs) for CT-optimal and nonoptimal touch

The ANOVA revealed a significant effect of Group (SPD, Control) for pleasure (F(1,130) = 24.61, p < 0.001, η^2^G = 0.109), arousal F(1,130) = 76.09, p < 0.001, η^2^G = 0.289), and the urge to perform skin-picking (F(1,130) = 99.90, p < 0.001, η^2^G = 0.352). Patients with SPD reported less pleasure (M_SPD_ = 5.02, SD = 1.19, M_Control_ = 6.12, SD = 1.36), higher arousal (M_SPD_ = 3.63, SD = 1.19, M_Control_ = 2.03, SD = 0.88), and a greater urge to pick their skin (M_SPD_ = 3.46, SD = 1.42, M_Control_ = 1.46, SD = 0.73) while being touched (Fig. 2).

The main effect for Type of Touch (CT-optimal/nonoptimal) was significant for pleasure (F(1,130) = 190.24, p < 0.001, η^2^G = 343), arousal F(1,130) = 35.54, p < 0.001, η^2^G = 0.077) and urge to perform skin-picking (F(1,130) = 13.28, p < 0.001, η^2^G = 0.029). CT-optimal touch was accompanied by more pleasure (M_nonoptimal_ = 4.40, SD = 1.76, M_optimal_ = 6.67, SD = 1.58), less arousal (M_nonoptimal_ = 3.25, SD = 1.69, M_optimal_ = 2.51, SD = 1.28), and less urge to pick one’s skin (M_nonoptimal_ = 2.76, SD = 1.83, M_optimal_ = 2.28, SD = 1.54) relative to CT-nonoptimal touch (Fig. 2). The interaction Group x Type of Touch was not significant (p > 0.06, η^2^G = 0.01).

### Discriminative touch

The two-point discrimination threshold did not differ between the SPD group (M = 3.07 mm; SD = 0.57) and the Control group (M = 3.10 mm; SD = 0.49); t_130_ = 0.34 (p = 0.734).

### Brain activity

#### Between-group findings

**Fig. 3:**
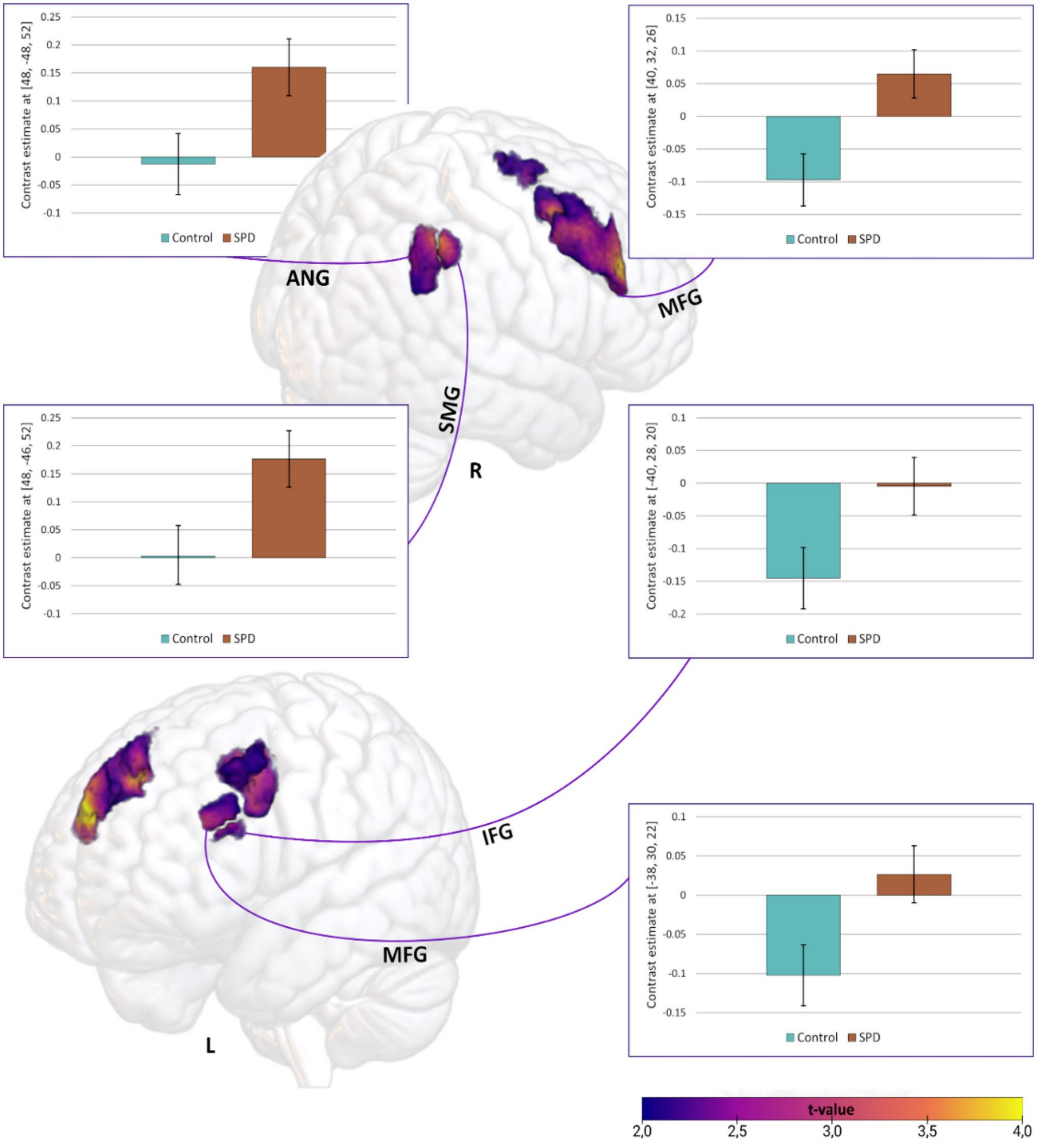
Comparison of brain activity between the SPD group and the Control group for the contrast CT-optimal touch vs. nonoptimal touch. *SPD* skin-picking disorder, *ANG* angular gyrus, *SMG* supramarginal gyrus, *IFG* inferior frontal gyrus, *MFG* medial frontal gyrus

For the contrast CT-optimal/nonoptimal touch, the SPD group was characterized by increased ROI activity in the right supramarginal gyrus (SMG) and the right angular gyrus (ANG; Table 2). The control group showed deactivation in prefrontal cortex regions (left/ right middle frontal gyrus (MFG), left inferior frontal gyrus; IFG), which was absent in the SPD group (Fig. 3). There were no significant effects on the whole-brain level. Within-group findings are displayed in Supplementary Table S1 and Figure S1.

**Table 2:** Comparison of activity in regions of interest between the SPD group and the Control group for the contrast CT-optimal touch–nonoptimal touch

	H	X	Y	Z	t	p(FWE)	Number of voxels
SPD > Control							
Middle frontal gyrus	L	− 38	30	22	3.98	0.037	87
Middle frontal gyrus	R	40	32	26	4.91	0.001	677
Inferior frontal gyrus	L	− 40	28	20	3.61	0.029	23
Angular gyrus	R	48	− 48	52	3.82	0.033	211
Supramarginal gyrus	R	48	− 46	52	3.84	0.027	88
Control > SPD							
No significant findings							

*SPD* skin-picking disorder, *H* hemisphere, *x,y,z* MNI coordinates, p corrected for family-wise error on the peak-level

### Exploratory functional connectivity analysis

Patients with SPD showed enhanced coupling between the right SMG and the right MFG (MNI coordinates: 34, 8,58; t = 4.07; p(FWE) = 0.033; cluster size = 85) and the right ANG and the left SMG (MNI coordinates: − 56, − 40, 32, t = 3.75, p(FWE) = 0.035; cluster size = 31) relative to the control group.

### Exploratory correlation analyses

#### SPD Group

Valence ratings were negatively correlated with activity in the right insula (MNI coordinates x,y,z: 42,0,2, t = 3.65, p = 0.031, cluster size = 22). The urge to pick one’s skin was negatively correlated with activity in the left SMG (MNI coordinates x,y,z: − 62, − 42,26, t = 3.99, p = 0.024; cluster size = 80).

#### Control group

Arousal ratings were positively correlated with activity in the right SMG (MNI coordinates x,y,z: 56,− 28,48, t = 3.65, p = 0.048; cluster size = 102) and left insula (MNI coordinates x,y,z: − 34, − 24,16. t = 4.04, p = 0.026, cluster size = 26). Valence ratings for soft touch were positively correlated with right insula activity (MNI coordinates x,y,z: 38,− 10,14, t = 3.63, p = 0.035, cluster size = 43).

## Discussion

This fMRI study investigated responses to caress-like touch in patients with skin-picking disorder (SPD). In the patient group, CT-optimal touch was accompanied by increased activation in the right supramarginal gyrus (SMG) and the angular gyrus (ANG). Both gyri are located in the posterior parietal cortex which functions as a multimodal integration area for somatosensory, auditory, and visual information. SMG activation has repeatedly been detected in studies on tactile processing [20, 28, 29]. The SMG is recruited during tactile exploration of surface texture and shape of objects [28, 29], when being touched by others, during self-touch, and the observation of touch [14, 20]. The SMG also contributes to gesture recognition and interpretation, an important area of nonverbal communication related to emotional processing [30]. In line with this, the right SMG appears to play a central role in self-other distinction and empathy (the ability to understand and share the feelings of others). In a study by Silani et al. [19], right SMG activity increased when participants had to make empathic judgments about another person’s affective states based on visual/tactile information.

The SMG together with the ANG is also involved in attention [31, 32]. Corbetta et al. [32] have suggested that both regions are part of the ventral attention network (VAN) that supports bottom-up attention to behaviorally relevant stimuli. The VAN also encompasses the middle/inferior frontal gyri (MFG, IFG). It has been suggested that the MFG functions as a circuit breaker that interrupts ongoing attentional processes. This region is involved in the flexible modulation of endogenous and exogenous attention (reorienting; e.g., [33]). Moreover, the IFG is involved in attentional control and response inhibition [34]. In the current study, only the control group displayed deactivation of the IFG/MFG during CT-optimal touch. This response might reflect that when being caressed, no attentional shifts and no motor actions are necessary. Caressing can be passively enjoyed by focusing on the pleasant sensation.

The present findings shed new light on tactile processing deficits in SPD. The clinical group did not show changes in tactile discrimination performance (two-point discrimination). Thus, pathological skin-picking did not affect skin sensitivity. However, dysfunctions referred to attentional processes in the context of being touched. The observed abnormalities in the attentional control network encompassed changes in localized activity (SMG, ANG, MFG, IFG), as well as connectivity (MFG-SMG; ANG–SMG). These findings illustrate that neural correlates of altered touch processing in SPD can be found both at the level of individual brain regions as well as a network level. Increased localized parietal ROI activation and reduced frontal ROI deactivation were accompanied by increased ROI coupling in the SPD group. A similar activity/connectivity pattern characterized a haptic discrimination task [41]. The participants of that study had to judge whether a touched shape or texture corresponded to a previously presented stimulus. In trials with a haptic mismatch (tactile information did not meet expectations), SMG/MFG activity increased as well as SMG connectivity. Unfortunately, the chosen gPPI approach of the present investigation does not provide information on how the ROIs directed their influence on each other (e.i., which region influenced the other region). Other methods, such as effective connectivity analyses, would be required to specify the observed SPD-related changes in functional integration and infer causality.

Moreover, the consequences of these neural alterations need further exploration. Patients with SPD may use intense self-stimulation of their skin to redirect their attention from external stressors to internal sensations. Typical elicitors of skin-picking episodes are negative affective states and bodily tension, which are reduced via repetitive skin manipulation [3]. Different authors (e.g., [16, 35]) have argued that BFRBs (pathological hair-pulling, skin-picking) may help individuals who feel overstimulated, to distract themselves from intense external stimulation. The BFRBs enable them to shut everything else out, bringing on an almost trance-like and self-absorbed state. On the other hand, weak stimulation or under-stimulation may also trigger BFRBs, which then have the function to establish sufficient arousal and well-being [35]. In line with this concept, the SPD group reported a greater urge to pick their skin while being gently touched and gave lower valence ratings than the control group. Moreover, an unusual correlation pattern emerged in the SPD group: greater insula activity was associated with less positive valence ratings for touch.

Interestingly, the role of sensory sensitivity and active sensing in the context of mental disorders has been widely neglected thus far [36]. For example, in the National Institute of Mental Health's Research Domain Criteria [RDoC] framework, these aspects appear to be either absent or underdeveloped. This is surprising since a multitude of research findings point to the presence of alterations in the processing of CT-optimal (affective) touch in various mental disorders, including diagnoses such as autism, autism spectrum disorders, eating disorders, and borderline personality disorder [10–12, 37]. Deficits in affective-touch processing are correlated with reduced buffering of stress symptoms [38], disorganized attachment style [39], and reduced psychological well-being [40]. Therefore, future research should continue to focus on the processing of tactile stimuli, and the accompanying emotional processes, in the context of skin-picking disorder.

### Limitations of the present study

All participants in the present study were female, which limits the generalizability of our results. At the same time, our sample may be representative of the female SPD population at large. Further, this study was neither designed nor powered to address the possible influences of comorbidities on brain activation. Nonetheless, exploratory analyses showed that comorbidity did not significantly impact brain activation in the SPD group. In addition, only three patients in the SPD group were taking psychotropic (antidepressant) medication.

## Conclusions

The present fMRI study detected altered activity and connectivity in the ventral attention network of patients with SPD while being gently touched.

### Supplementary Information

Below is the link to the electronic supplementary material.

Supplementary file1 (DOCX 701 KB)

## Data Availability

Data are available from the corresponding author.
