# Supplementary material for: Brain mechanisms for processing caress-like touch in skin-picking disorder
Source: Eur Arch Psychiatry Clin Neurosci. 2023 Aug 23;274(1):235–43. doi: 10.1007/s00406-023-01669-9 (PMC10786990; doi:10.1007/s00406-023-01669-9)

**Supplementary Table S1: Within-group findings**

|  |  |  |  |  |  |  |  |
| --- | --- | --- | --- | --- | --- | --- | --- |
|  | **H** | **x** | **y** | **z** | **t** | **p(FWE)** | **Cluster size** |
| **Within SPD group: CT-optimal > non-optimal touch/ ROI findings** | | | | | | | |
| Angular gyrus | L | -44 | -52 | 56 | 5.01 | 0.0009 | 40 |
| Angular gyrus | R | 46 | -46 | 56 | 6.32 | <0.0001 | 362 |
| Inferior frontal gyrus | R | 56 | 12 | 24 | 5.26 | 0.0003 | 44 |
| Middle frontal gyrus | L | -32 | -4 | 62 | 7.11 | <0.0001 | 83 |
| Middle frontal gyrus | R | 46 | 2 | 54 | 5.62 | 0.0003 | 302 |
| Supramarginal gyrus | L | -46 | -44 | 56 | 7.03 | <0.0001 | 122 |
| Supramarginal gyrus | R | 44 | -40 | 54 | 7.98 | <0.0001 | 323 |
| **Whole brain findings** |  |  |  |  |  |  |  |
| Supramarginal gyrus | R | 48 | -26 | 40 | 12.42 | <0.0001 | 19532 |
| Temporal occipital | R | 32 | -46 | -26 | 8.56 | <0.0001 | 2310 |
| Lateral occipital | L | -42 | -70 | 0 | 8.05 | <0.0001 | 1126 |
| Cerebellum | R | 22 | -54 | -54 | 7.98 | <0.0001 | 1058 |
| Putamen | L | -24 | -4 | 4 | 7.17 | 0.0001 | 467 |
| Occipital fusiform | L | -16 | -70 | -22 | 5.86 | 0.0081 | 642 |
| **Within SPD group: CT-nonoptimal > CT-optimal/ ROI findings** | | | | | | | |
| Inferior frontal gyrus | L | -52 | 16 | 14 | 4.67 | 0.0021 | 443 |
| Inferior frontal gyrus. | R | 52 | 18 | 8 | 3.59 | 0.0442 | 104 |
| Insula | L | -40 | -2 | -14 | 6.93 | <0.0001 | 441 |
| Insula | R | 36 | -20 | 18 | 7.55 | <0.0001 | 290 |
| **Whole brain findings** |  |  |  |  |  |  |  |
| Postcentral gyrus | R | 36 | -26 | 60 | 15.50 | <0.0001 | 1207 |
| Precentral gyrus | R | 36 | -22 | 20 | 8.72 | <0.0001 | 11459 |
| Cerebellum | L | -2 | -46 | -58 | 5.51 | 0.0272 | 442 |
| **Within Control group: CT-optimal > nonoptimal touch/ ROI findings** | | | | | | | |
| Angular gyrus | L | -48 | -62 | 16 | 5.07 | 0.0009 | 73 |
| Angular gyrus | R | 50 | -52 | 12 | 7.17 | <0.0001 | 230 |
| Inferior frontal gyrus | R | 56 | 12 | 24 | 6.54 | <0.0001 | 56 |
| Middle frontal gyrus | L | -28 | -6 | 54 | 7.64 | <0.0001 | 63 |
| Insula | L | -34 | -6 | 12 | 4.36 | 0.0103 | 43 |
| Insula | R | 34 | -6 | 12 | 4.64 | 0.0045 | 39 |
| Middle frontal gyrus | R | 32 | -4 | 58 | 4.76 | 0.0062 | 23 |
| Supramarginal gyrus. | L | -44 | -44 | 52 | 4.26 | 0.0122 | 20 |
| Supramarginal gyrus. | R | 38 | -38 | 46 | 8.95 | <0.0001 | 143 |
| **Whole brain findings** |  |  |  |  |  |  |  |
| Precentral | L | -24 | -12 | 58 | 13.22 | <0.0001 | 18766 |
| Middle temporal gyrus | R | 44 | -58 | 10 | 11.01 | <0.0001 | 4383 |
| Lateral occipital inferior | L | -40 | -62 | 6 | 9.75 | <0.0001 | 3411 |
| Cerebellum | R | 26 | -58 | -52 | 7.88 | <0.0001 | 1736 |
| Putamen | L | -28 | -12 | 10 | 7.70 | <0.0001 | 1176 |
| Insula | R | 32 | -8 | 10 | 5.62 | 0.0260 | 436 |
| Frontal pole | L | -2 | 56 | -2 | 5.55 | 0.0323 | 2005 |
| **Within Control group: CT-nonoptimal > optima touch/ ROI findings** | | | | | | | |
| Inferior frontal gyrus | L | -48 | 18 | 18 | 5.20 | 0.0005 | 405 |
| Inferior frontal gyrus | R | 52 | 18 | 6 | 4.96 | 0.0009 | 146 |
| Middle frontal gyrus | L | -38 | 30 | 20 | 5.16 | 0.0019 | 289 |
| Middle frontal gyrus | R | 40 | 34 | 42 | 4.56 | 0.0114 | 285 |
| **Whole brain findings** |  |  |  |  |  |  |  |
| Precentral gyrus | R | 34 | -26 | 66 | 14.37 | <0.0001 | 1067 |
| Cerebellum | L | -10 | -50 | -16 | 6.53 | 0.0013 | 3147 |
| Frontal operculum | R | 40 | 24 | 8 | 6.30 | 0.0028 | 2572 |
| Inferior frontal gyrus | L | -54 | 26 | 22 | 5.55 | 0.0321 | 2577 |

Footnote: H: hemisphere, x,y,z: MNI coordinates; p corrected for family-wise error (FWF); t: t-value; SPD skin-picking disorder; ROI: region of interest

**Supplementary Figure S1: Within-group findings**


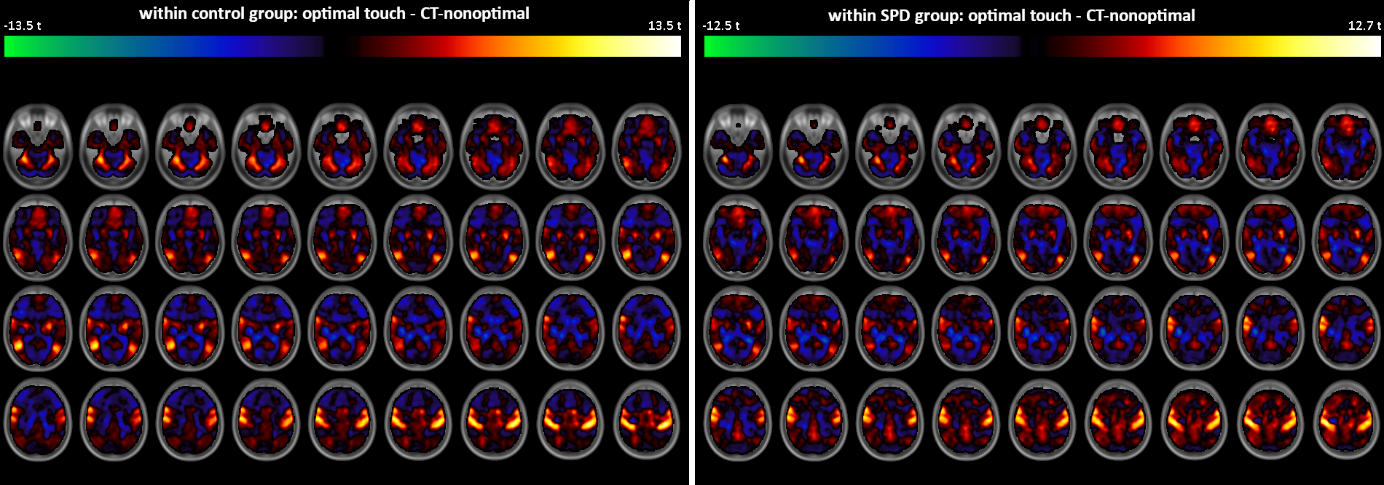

Supplement: Supplementary file 1 — Supplementary file1 (DOCX 701 KB) [file 406_2023_1669_MOESM1_ESM.docx]
